# Unveiling the combined effects of neutral dynamics and electrodynamic forcing on dayside ionosphere during the 3–4 February 2022 “SpaceX” geomagnetic storms

**DOI:** 10.1038/s41598-023-45900-y

**Published:** 2023-11-02

**Authors:** Geetashree Kakoti, Mala S. Bagiya, Fazlul I. Laskar, Dong Lin

**Affiliations:** 1 Indian Institute of Geomagnetism (DST), Navi Mumbai, India; 2 Laboratory for Atmospheric and Space Physics, University of Colorado, Boulder, CO USA; 3 High Altitude Observatory, National Center for Atmospheric Research, Boulder, CO USA

**Keywords:** Space physics, Space physics

## Abstract

Geomagnetic storms of G1-class were observed on 3 and 4 February 2022, which caused the loss of 38 out of 49 SpaceX satellites during their launch due to enhanced neutral density. The effects of storm-time neutral dynamics and electrodynamics over the American sector during this minor storm have been investigated using Global Positioning System—total electron content (TEC) and Global‐scale Observations of the Limb and Disk (GOLD) mission measured thermospheric composition and temperature. Results revealed an unexpected feature in terms of increase in O/N_2_ and depletion in TEC over the American low-latitudes. This feature is in addition to the classic storm time ionospheric variations of enhancement in ionospheric electron density in presence of enhanced O/N_2_ and an intense equatorial electrojet (EEJ). Further, significant morning-noon electron density reductions were observed over the southern mid-high latitudes along the American longitudes. Results from Multiscale Atmosphere-Geospace Environment (MAGE) model simulations elucidated storm-induced equatorward thermospheric wind which caused the strong morning counter electrojet by generating the disturbance dynamo electric field. This further explains the morning TEC depletion at low-latitudes despite an increase in O/N_2_. Sub-storm related magnetospheric convection resulted in significant noon-time peak in EEJ on 4 February. Observation and modelling approaches together suggested that combined effects of storm-time neutral dynamic and electrodynamic forcing resulted in significant ionospheric variations over the American sector during minor geomagnetic storms.

## Introduction

A powerful eruption from the AR2936 sunspot released an M1-class solar flare on 29 January 2022 and an asymmetric halo Coronal Mass Ejection (CME) series. The flare was a long-duration flare of 4 h, thus inducing more energy to the accompanying CME that hit the Earth's magnetic field on 1 February 2022 at ~ 22:21 UT (https://www.spaceweather.com). Nevertheless, its initial impact did not cause any immediate storm effects but caused weak geomagnetic storms on 3 and 4 February, which continued till 5 February.

It is speculated that minor geomagnetic activity started on 3 February caused significant neutral atmospheric changes at very low earth orbit (VLEO) altitude^1–5^. SpaceX launched 49 Starlink satellites into their initial orbit of ~ 210 km at 18:13 UT on 3 February from Kennedy Space Center in Florida. The satellites were supposed to be lifted using electrical thrusters to their operational level of 550 km^1,6^*.* If the reports from SpaceX are to be true, then minor storm activity that occurred during 3–4 February enhanced the atmospheric drag by more than 50% and resulted in the loss of 38 Starlink satellites (from https://www.spacex.com/updates/).

The geomagnetic storm-induced ionospheric-thermospheric (I-T) disturbances are primarily driven by the penetration of storm time electric fields from high to low-latitudes, heating up of the auroral zone, which leads to enhanced thermospheric circulation, subsequent delayed generation of disturbance dynamo electric field, and changes in the atmospheric composition^7–15^. The low-latitude ionosphere is highly sensitive to the direct penetration of storm time magnetospheric convective field from high-latitudes, generation of disturbance dynamo electric field, and thermospheric neutral composition changes^15–26^. Numerous studies have been performed to understand the I-T response to intense and super geomagnetic storms. Some of such important studies include the I-T response to geomagnetic storms that occurred during solar cycles 23 and 24; e.g., 24 November 2001 storm^27,28^, 29 October 2003 Halloween Storm^18,29–32^, 8 November 2004 storm^33,34^,15 May 2005 storm^15,35^ and most recent 17 March 2015 and 22–23 June 2015 storms^24,26,36–45^.

At the other end, the I-T variations during moderate to minor geomagnetic storms received less attention. It should be noted that the occurrence frequency of moderate to minor storms is much higher than the intense storms. Despite this, a very less has been explored on low-latitude ionospheric changes during moderate to minor storm activity. This could be probably due to the less severity of the effects they produce and thus it might be difficult to distinguish the storm-induced changes in respect to the background variations^46–48^. However, the recent incidence of the loss of 38 out of 49 SpaceX satellites which brought an economic loss estimated to be several tens of millions of dollars^1,6^, alerted the community to look into the I-T perturbations during minor storm activity also^1–5,49,50^. Considering the lesson learned from this incident, SpaceX changed its launch procedures in the next Starlink launch on 21 February by changing the initial orbits to a higher height of 300 km^1^.

The present study is all about analyzing the dayside I-T changes during the G1 class storms that occurred on 3–4 February 2022. Our analysis based on the Global Positioning System (GPS) measured Total Electron Content (TEC) over the American longitudes suggested substantial variations in ionospheric electron density at low-mid latitudes with significant hemispheric asymmetry during 3–4 February 2022. These variations are presented and discussed in terms of the storm time electrodynamical and neutral dynamical changes. The equatorial electrojet (EEJ) derived using ground-based magnetometer data and GOLD measured O/N_2_ respectively provided insights into electrodynamical and neutral dynamical changes over the American longitudes during this minor geomagnetic storm activity. The Multiscale Atmosphere-Geospace Environment (MAGE) model derived thermospheric winds and vertical plasma drift along with the Global‐scale Observations of the Limb and Disk (GOLD) measured thermospheric temperature explains the physical mechanism behind the observed variations. It is believed that the present study would be useful to understand the high- to low-latitude interactions during minor geomagnetic storms and their multifaceted effects at low latitudes.

## Results

### Interplanetary and geomagnetic conditions

**Figure 1:**
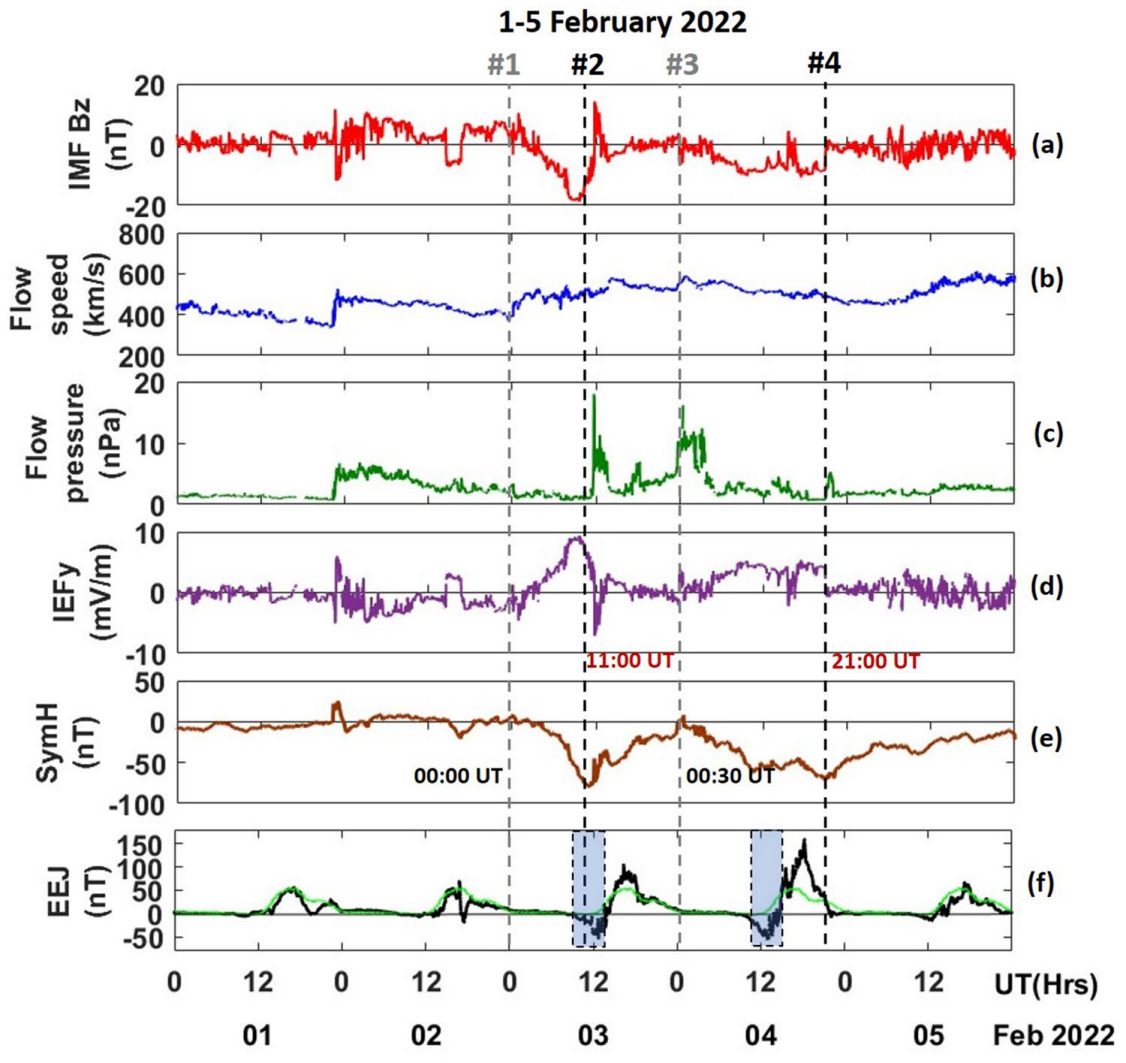
(**a**) North–South component of interplanetary magnetic field (IMF Bz), (**b**) solar wind speed, (**c**) solar wind flow pressure, (**d**) interplanetary electric field (IEFy), (**e**) symmetric ring current index (SYM-H) and (**f**) equatorial electrojet (EEJ) over American sector, for the period of 1–5 February 2022.

Figure 1a–f presents interplanetary and geomagnetic parameters during 1–5 February 2022. The halo CME erupted from the sunspot AR2936 hit the Earth's magnetic field on 1 February 2022 at ~ 22:21 UT. This resulted in sudden rise in the solar wind speed and flow pressure at ~ 22:23 UT, which was accompanied by a short excursion in IMF Bz from northward to southward (Fig. 1a). Due to the sudden intrusion of CME shock indicated by the sudden rise in the solar wind flow speed (~ 525 km/s) and pressure (~ 5.5 nPa), the magnetosphere was compressed, causing a sudden storm commencement (SSC) on 1 February (Fig. 1b, c). The SSC is characterized by intensification in the ring current intensity represented by an abrupt rise in the SYM-H index to 25 nT at ~ 23:05 UT (Fig. 1e). However, the main phase of the 03 February geomagnetic storm started at ~ 00:00 UT (vertical dashed line #1 in Fig. 1), as seen from the SYM-H. The sharp reduction in the SYM-H value reaching a minimum of − 80 nT occurred at ~ 11:00 UT (vertical line #2 in Fig. 1), resulting in a G1-class geomagnetic storm. This was a minor storm (Kp index ≤ 5). The solar wind flow pressure sharply increased to ~ 18 nPa at ~ 11:44 UT (Fig. 1c). IMF Bz turned southward at ~ 03:58 UT and remained in the same direction till about ~ 11:47 UT on 3 February. The IMF Bz turned northward with a temporary excursion during 11:48–12:44 UT.

The dawn to dusk component of interplanetary electric field (IEFy) (derived as *Electric field* (mV/m) = − *V*(km/s) × *Bz*(*nT*; *GSM*) × 10^–3^), which is proxy for the electric field over the low-latitude showed a sharp increase and decrease during the southward and northward turning of IMF Bz (Fig. 1d). A decrease in SYM-H was observed again at ~ 00:30 UT on 4 February (vertical line #3 in Fig. 1), reaching a minimum of ~ − 70 nT at ~ 21:00 UT on 4 February (vertical line #4 in Fig. 1). The storm recovery coincided with a stream of high-speed solar wind on the 5 and 6 February, with a maximum solar wind speed of ~ 616 km/s (Fig. 1b).

Figure 1f shows the equatorial electrojet variation during 1–5 February 2022 over the American sector. The green line indicates the quiet days average of EEJ. EEJ variations exhibited anomalous behaviour on 2 February, and its development coincided with the sudden southward turning of IMF Bz and increased in IEFy (14:00–16:42 UT). On 3 February, EEJ development was significantly perturbed and characterized by the development of strong Counter Electrojet (CEJ) during 9:00–14:30 UT, with a minimum magnitude of ~ − 51 nT at ~ 12:46 UT. A sharp decrease at ~ 11:40 UT coincided with the northward turning of IMF Bz and a sharp reduction in IEFy. This was followed by a strong increase in the EEJ strength, reaching a maximum value of ~ 103 nT at ~ 16:36 UT. A similar variation was observed on 4 February, CEJ occurred during 9:00–15:00 UT with peak magnitude ~ − 55 nT at ~ 13:13 UT, followed by strong overall enhancement in EEJ from 15:00 to 21:30 UT with peak magnitude ~ 158 nT at ~ 18:25 UT. The occurrence of CEJ on both 3 and 4 February is attributed to the presence of disturbance dynamo electric field, which will be discussed later. On 5 February magnitude of EEJ recovered to its normal level, and the appearance of CEJ almost vanished.

### Ionospheric and thermospheric conditions on 3 and 4 February 2022

**Figure 2:**
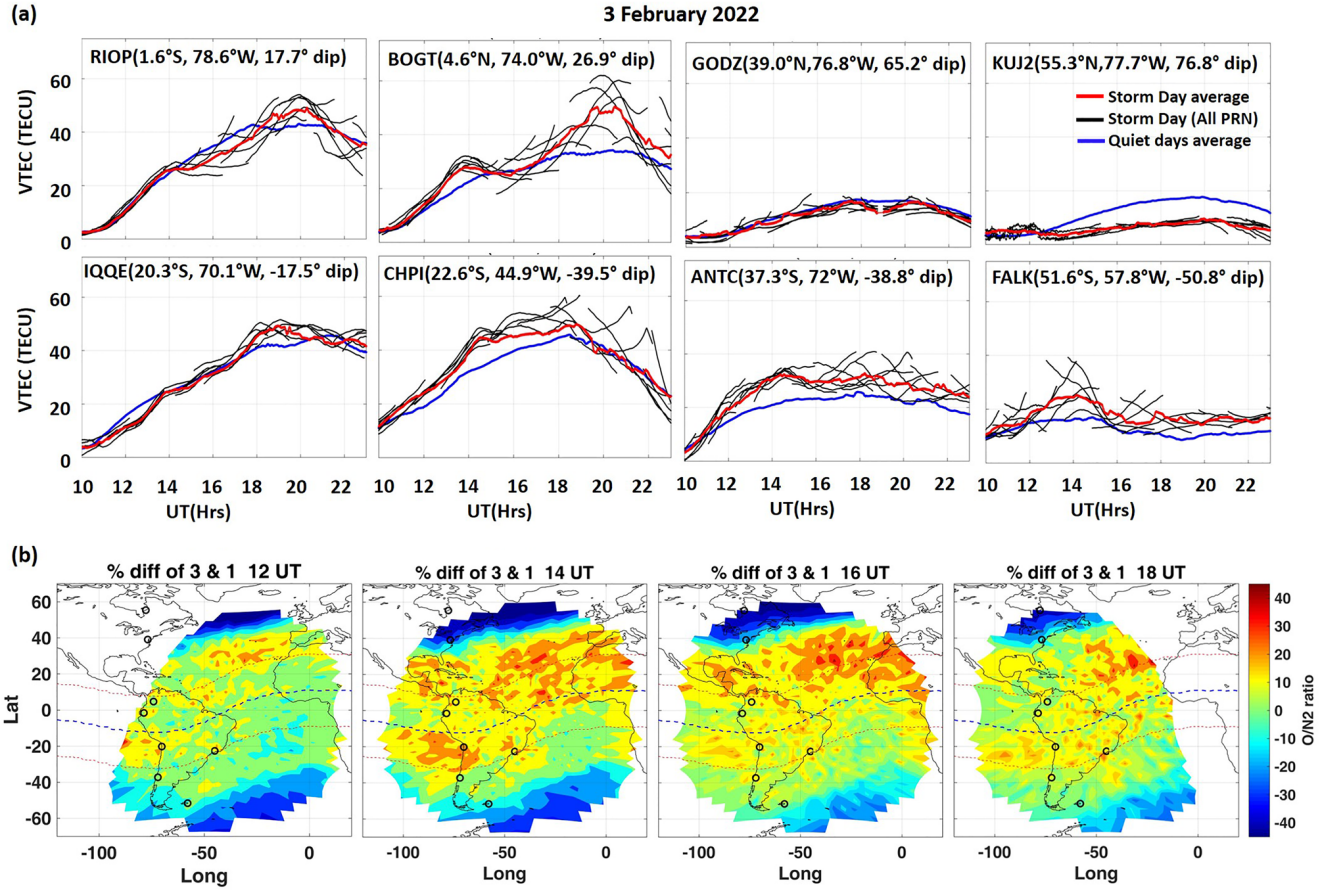
(**a**) TEC variations during 10:00–23:00 UT over the northern hemisphere (NH) (upper panel) and Southern hemisphere (SH) (lower panel) at low, mid and high latitude stations on 3 February 2022 for the American sector. (**b**) ΣO/N_2_ changes (%Diff) from GOLD observations on 3 February from 1 February. Black circles indicate the GPS-TEC stations.

Figure 2 presents the daytime I-T variation on 3 February 2022. The diurnal TEC variations over the American longitudes were analyzed for each hemisphere. The daytime (10:00–23:00 UT) TEC variations from low, mid and high-latitude stations over the northern hemisphere (NH) (upper panel) and southern hemisphere (SH) (lower panel), are depicted in Fig. 2a. The storm days TEC are presented along with the quiet day average (blue line) for each station. TEC observations on the five quietest days of the month before and after the storm days (WDC, Kyoto; http://wdc.kugi.kyoto-u.ac.jp/) were calculated and averaged at every 1-min.

The TEC exhibited distinct storm-time perturbations over all latitudes. During the recovery phase on 3 February, storm-time TEC variation was similar to quiet time over the low-latitude station RIOP in NH (geomag. latitude: 7.7°N; LT = UT-5.2). However, a strong daytime positive response in TEC was observed near EIA crest station BOGT (geomag. latitude: 14°N; LT = UT-4.9), from 17:30 to 22:00 UT (Fig. 2a). Over BOGT, the rise in TEC was noticed from ~ 12:00 UT reaching a peak at ~ 13:30 UT and then decreased abruptly till ~ 17:30 UT after which a strong daytime enhancement persisted. Storm-time perturbations were not apparent in the mid-latitude station GODZ (geomag. Latitude: 48.2°N; LT = UT-5.1). While, over the NH high-latitude station KUJ2 (geomag. latitude: 64.5°N; LT = UT-5.2), a strong negative response in TEC was noticed (~ -20 TECU, i.e. > 80%). The depletion in high-latitude TEC is generally attributed to the decrease in O/N_2_ ratio as a result of the upwelling in the auroral region due to storm-time auroral heating^51,52^*.* The upwelling cause oxygen-depleted or nitrogen-rich air to move up to height of F region, which enhances the ion recombination that either reduce the rate of plasma density increase or can cause a decrease in plasma density^53^. During the main and recovery phases on 3 February, TEC showed a moderate positive response over the southern middle and high-latitude stations. Over the SH mid and high-latitude stations, ANTC (geomag. latitude: 28°S; LT = UT-4.8) and FALK (geomag. latitude: 42.6°S; LT = UT-3.8), slight enhancement in TEC can be seen throughout the recovery phase of the storm (12:00–23:00 UT). Storm-time TEC perturbations were not so significant over low-latitude station IQQE (geomag. latitude: 10.8°S; LT = UT-4.6), whilst minor enhancement was seen during the early recovery period (11:00–18:00 UT) over the near-crest location CHPI (geomag. latitude: 14.26°S; LT = UT-3). The mid-high latitude TEC enhancement during the minor storm, which is not a usual storm-time characteristic, could be contributed by the storm-time changes in thermospheric circulations. The high-latitude depletion and low-latitude enhancement in TEC and O/N_2_ during the storm period were reported earlier^54^. A very few studies have reported O/N_2_ enhancement in mid-high latitudes during geomagnetic storms^40,55–57^. During the storm main-phase, positive dayside ionospheric response, or the storm-enhanced density (SED) arises at high and mid-latitudes could be due to the uplift of plasma to higher altitudes with lower recombination rate and horizontal plasma transport^58,59^.

During a geomagnetic storm, the thermospheric circulation is perturbed by the enhanced joule heating caused by strong high-latitude electric current and ion-neutral collisions. This disturbed circulation can drive large changes in the thermospheric temperature and composition at lower latitudes^52,60^. Recent studies using GOLD-derived O/N_2_, Oxygen-I (OI) 135.6 nm emission, and limb and disk temperature showed that weak geomagnetic storms could significantly affect the thermosphere-ionosphere system^5,61–65^*.* Figure 2b depicts the percentage difference (% Diff) of GOLD measured ΣO/N_2_ on 3 February from 1 February in both hemispheres. Each vertical panel is respectively for 12, 14, 16, and 18 UT. The black circles indicate the position of TEC stations. The percentage difference (% Diff) of ΣO/N_2_ between the reference quiet day and storm days is calculated by subtracting the quiet time ΣO/N_2_ values from the storm time values and then dividing by the quiet time values for a given UT. For the current storm, 1 February is considered as the reference quiet day with an average kp index ≤ 1. Here, we consider the nearest quiet day to avoid the seasonal effects.

Clear storm-time perturbations were observed in ΣO/N_2_. Low-latitude enhancement and mid to high-latitude depletion in ΣO/N_2_ were observed during the recovery phase on 3 February. A very strong enhancement was observed between ∼ 40°W and ∼ 90°W from ∼ 10°S to ∼ 40°S during ~ 12:00–18:00 UT with a maximum enhancement of ~ 40% at ~ 14:00 UT. In contrast, significant depletion can be seen between ∼ 50°W and ∼ 0°W from ∼ 50°S to ∼ 80°S during ~ 12:00 and ~ 16:00 UT, with maximum depletion of ∼ 32%. However, between ∼ 60°W and ∼ 90°W, strong depletion was only observed at high-latitudes. Similarly, over the northern hemisphere, significant enhancement at low to mid-latitudes (from ∼ 0 to 40°N) and depletion in the high-latitudes in ΣO/N_2_ was observed on 3 February with maximum enhancement of ~ 45% and depletion of ~ − 58%. The low-mid latitude enhancement was more prominent before noon (~ 14:00 and 16:00 UT) over the SH. The storm time variation in ΣO/N_2_ was comparatively stronger over the NH compared to the SH (maximum percentage difference ≥  ± 45%), which is clearly reflected in the TEC variation (Fig. 2a). Thus, it could be stated that thermospheric variations during the storm period were clearly manifested in dayside TEC on 3 February.

**Figure 3:**
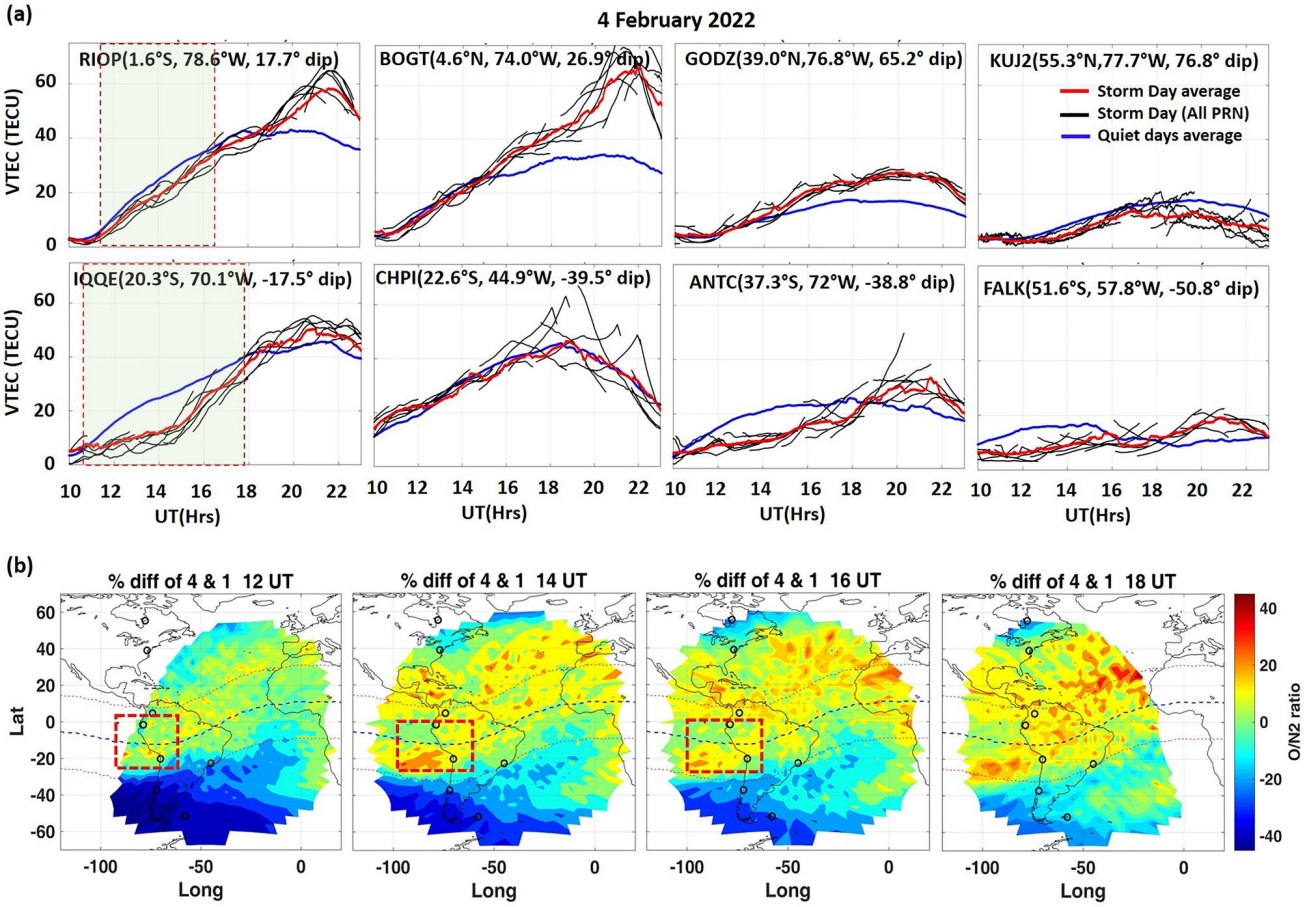
(**a**) TEC variations during 10:00–23:00 UT over northern hemisphere (NH) (upper panel) and Southern hemisphere (SH) (lower panel) low, mid and high latitude stations on 4 February 2022. (**b**) ΣO/N_2_ changes (%Diff) from GOLD observations on 4 February from 1 February. Black circles indicate the GPS-TEC stations. The Red dashed highlighted region indicates the unexpected feature i.e., an increase in O/N_2_ was accompanied by depletion in TEC during the morning.

Figure 3 shows the ionosphere-thermosphere variation on 4 February 2022. Figure descriptions are the same as given for Fig. 2. On 4 February, when geomagnetic activity resurged (0:00–21:00 UT) with SYM-H reaching a minimum of ~ − 62 nT, a strong daytime positive ionospheric response was observed over the northern low-latitudes. Substantial enhancement in daytime TEC was evident over the NH low-latitude station RIOP from 19:00 to 23:00 UT. Over BOGT, the peak enhancement of ~ 36 TECU (~ 116%) was observed at ~ 21:52 UT (~ 17 LT). The TEC enhancement was extended towards the mid-latitude stations indicating the poleward extension of EIA on the storm day. However, the magnitude of TEC enhancement decreased towards the higher latitude stations GODZ (~ 10.05 TECU at ~ 21:00 UT). At the high-latitude station, the negative effect persisted on this day, with maximum depletion by ~ − 7 TECU (~ 40%) at ~ 21:00 UT.

Over the southern hemisphere, after the growth of 2nd main phase on 4 February, the I-T condition became completely different from the previous day. Minor daytime enhancements were seen over SH stations during 19:00–23:00 UT except at low-latitude station CHPI. In contrast, the negative effect prevailed over all the SH stations from morning to noon hours except at CHPI. The depletion in TEC was seen from 11:00 to 18:00 UT over low-latitude station IQQE, and over the mid to high-latitude stations ANTC and FALK from 11:00 to 20:00 UT and 10:00–19:00 UT, respectively. This was followed by minor enhancement during evening hours. Thus, a clear morning-afternoon asymmetry exists in storm-time TEC over SH stations (except at CHPI).

The map for dTEC (TEC_disturbed day_ − TEC_quiet day_) variations over American sector on 3–4 February 2022 obtained from the Madrigal database are presented in Supplementary Fig. S2 for more information. Significant enhancement in NH low-latitudes and strong negative effect in NH high-latitude during daytime ionosphere could be clearly noticed from the figure. The positive afternoon effect in TEC continued on 4 February over both the hemispheres and with higher magnitude and latitudinal extension over NH. Thus, the poleward extension of EIA on the storm day was noticed over NH. In contrast, a strong negative effect was seen over the SH from morning to noon hours on 4 February (Supplementary Fig. S2).

The storm time negative/positive TEC perturbations are mainly caused by electrodynamical and/or neutral-dynamical changes (e.g., Bagiya et al.^15^ and references cited therein). The thermospheric composition was significantly disturbed on 4 February as observed from GOLD measure O/N_2_ (Fig. 3b). Over NH, the positive effect was more prevalent, and the negative impact was only limited to the high-latitudes, which is also reflected in the TEC variation over the NH. The storm time depletions in TEC and O/N_2_ at high-latitudes and enhancements at low/mid-latitudes are very well known. Over the SH, the negative effect was more prevalent, and a very strong depletion was observed from ∼ 30°S to ∼ 80°S over the whole longitude sector during ~ 12:00 and 14:00 UT. The morning depletion was more prevalent than the afternoon depletion. This could lead to the observed pre-noon depletion in TEC over the SH mid to high-latitudes (Fig. 3a). Over the SH low latitudes, enhancement in the ΣO/N_2_ was observed with maximum enhancement between ∼ 75°W and ∼ 100°W from ∼ 15°S to ∼ 25°S during ~ 14:00 UT. Thus, a strong interhemispheric asymmetry exists in the storm time variation of ΣO/N_2_. This asymmetry in the thermospheric composition perhaps resulted in observed interhemispheric asymmetry in TEC on 4 February. The severe negative/positive storm-time effect on I-T system over summer/winter hemisphere is a usual storm-time characteristic during intense storms. These variations are contributed by storm-time modification in the seasonal circulation patterns^66^. But what is new in this investigation is that it is a minor storm and could cause substantial storm-time seasonal behavior. Additionally, longitudinal difference was also observed in ΣO/N_2_ as there was a positive impact towards the east of ∼ 0°W from mid to high-latitudes. Whereas, over the northern hemisphere, a moderately positive impact was seen over the entire longitudinal range.

This investigation also brings forth some distinct/previously unseen results. The %Diff in O/N_2_ on 4 February shows a strong positive patch (15–30%) over the low-latitudes (Fig. 3b, red dashed highlighted region) that also includes the location of GNSS-receiver stations RIOP and IQQE. This enhanced ΣO/N_2_ suggests that TEC should also be enhanced on that day at that location. But in contrast, the TEC showed substantial depletion from ~ 11 to 18 UT, particularly over SH station IQQE and a minor depletion from ~ 11 to 16 UT over RIOP (see Fig. 3a, red highlighted region in 1st column), which is unexpected and puzzling. Typically, enhancement in TEC is caused by the increase in O/N_2_, since the electrons production in the ionosphere is directly linked to the photoionization of neutral atomic oxygen, and the reaction between electrons and molecular nitrogen is dominant loss process^51,67^. To study this unexpected behavior and the local electrodynamics, we looked into EEJ data. There was a strong morning CEJ event from 9:00 to 14:30 UT on 3 and 4 February, which was responsible for the TEC depletion over the low-latitudes despite of the presence of enhanced background O/N_2_.

Generally, CEJ during quiet days could be caused by changes in the atmospheric tides that control the global wind system at ionospheric heights. During this storm, the occurrence of CEJ events was most likely, related to the overshielding effect due to the northward turning of IMF Bz and disturbance dynamo electric (DDE) field that are usually observed during the recovery phase period, as explained in the discussion section. The generation of morning CEJ on 3 and 4 February due to disturbance dynamo is discussed in the following section.

### Neutral wind contribution in storm-time electrodynamics

**Figure 4:**
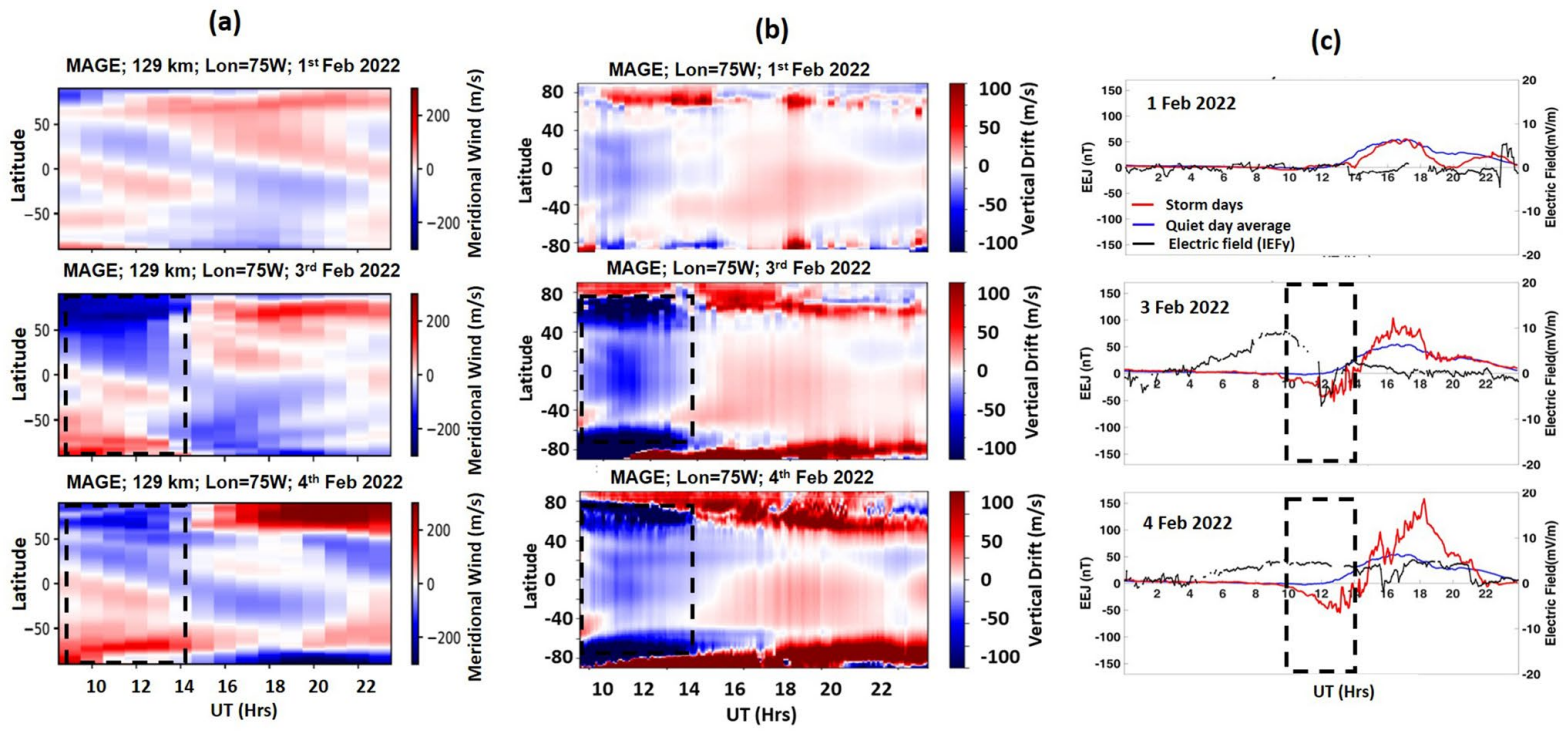
(**a**) Meridional neutral wind, and (**b**) vertical drift simulated by MAGE showed the development of strong equatorward wind and downward drift on both 3 and 4 February from 9:00 to 14:00 UT (black dashed highlighted region), (**c**) EEJ variations during 1, 3 and 4 February. Blue line indicates the quiet days average. Black line shows the dawn-to-dusk interplanetary electric field (IEFy). The black dashed highlighted regions indicate the presence of CEJ event.

The meridional neutral wind, and vertical drift simulated by MAGE on 1, 3 and 4 February 2022 from 9:00 to 23:00 UT are presented in Fig. 4a and b, respectively. Figure 4c depicts the EEJ variations during 1, 3 and 4 February along with the dawn-to-dusk interplanetary electric field (IEFy). As mentioned, the IEFy (derived as *Electric field* (mV/m) = − *V*(km/s) × *Bz*(*nT*; *GSM*) × 10^–3^) represents the proxy for the electric field over the low-latitude.

The MAGE simulated meridional neutral wind shows development of strong equatorward wind on both 3 and 4 February from ~ 9:00 to 14:00 UT (black dashed highlighted region). This storm time equatorward wind eventually drives the counter electrojet near equatorial region due to the generation of westward disturbance dynamo electric field. During geomagnetic storm, the additional energy input into the high latitude ionosphere results in Joule heating that drives the disturbance thermospheric winds above ~ 120 km which are equatorward at mid-latitudes. This interesting feature is captured by the GOLD-measured thermospheric disk temperature (Tdisk) which showed strong morning-afternoon asymmetry (Supplementary Fig. S3). A significant enhancement in thermospheric temperature was observed over mid-high latitudes during the local morning. The enhanced thermospheric temperature gives rise to the generation of equatorward meridional wind over mid-high latitudes.

Under the action of Coriolis force, a westward momentum is imparted to this equatorward circulation at mid-latitudes which give rise to a poleward electric field, westward E × B drift, and an eastward current. Based on the strength of wind and the global conductivity variations, this mid-latitude eastward current closes partly in the lower latitudes which generates a westward field at low-latitudes that opposes the normal Sq currents^8^*.* The model simulated zonal wind shows the development of westward wind at the mid-latitudes as a manifestation of equatorward neutral wind (black dashed highlighted region in Fig. 4a). This westward wind following the process of disturbance dynamo give rise to westward current at the low-latitudes that drives the observed westward equatorial electric field (CEJ) from ~ 9:00 to 14:30 UT on both 3 and 4 February. Thus, the model simulated winds clearly demonstrate the mechanism for development of morning westward field in the equatorial region. Furthermore, this westward electric field explains the unusual morning TEC depletion over the southern crest stations although there was an enhancement in background O/N_2_. Thus, this provides an explanation of the observed unexpected behaviour, and suggests that pre-noon ionosphere was primarily controlled by the storm time electrodynamical changes driven by DDE field rather than the composition changes during the storm condition of 4 February. Due to the lack of rich information on local time variations of O/N_2_ from earlier missions, such unexpected features were not observed or explained earlier. But GOLD being in a geostationary orbit provided good local time coverage to observe and explain such unexpected behaviours.

### Substorm contribution in storm-time electrodynamics

**Figure 5:**
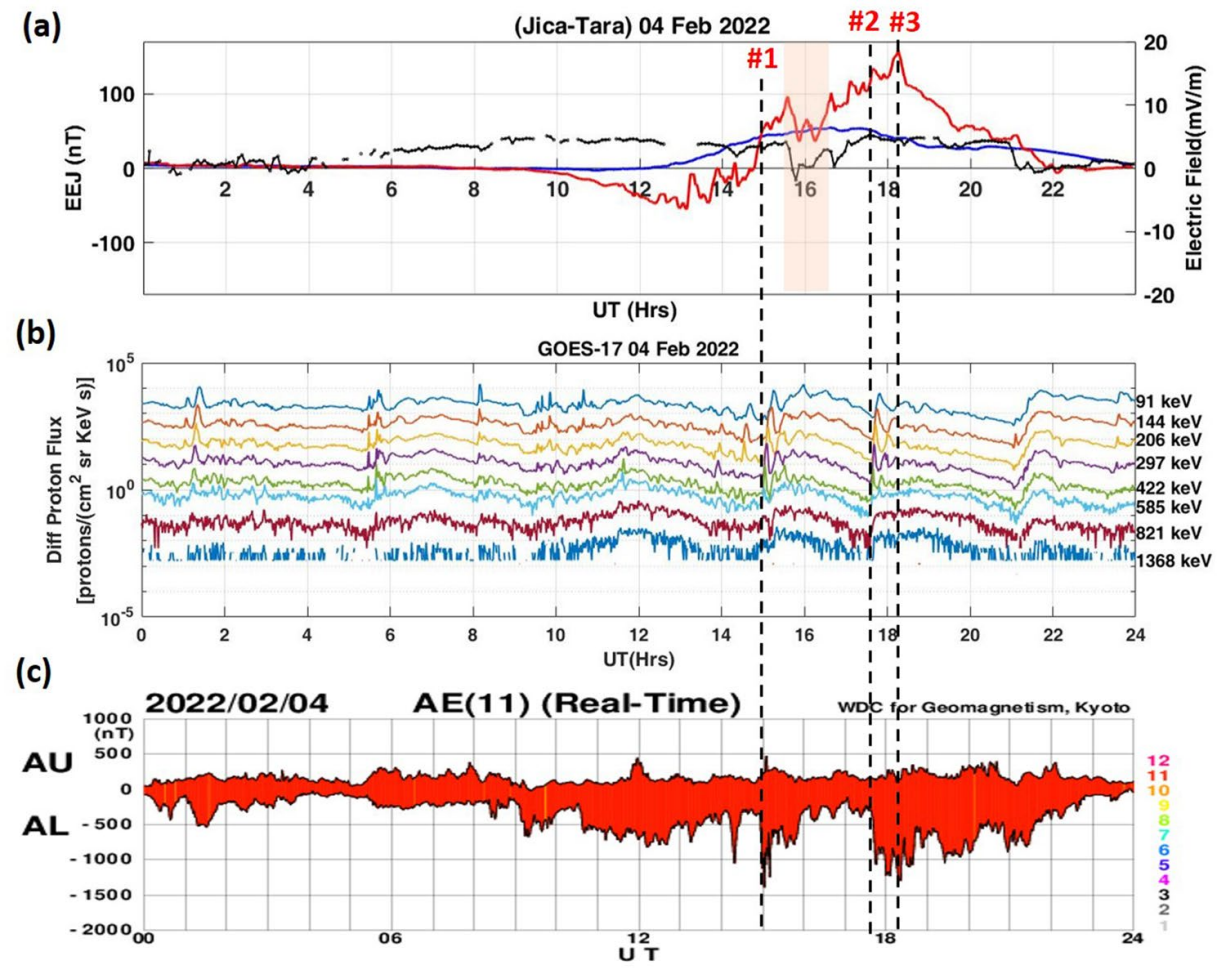
(**a**) EEJ and IEFy variations (**b**) GOES-17 derived Differential Proton Flux (**c**) AL and AU indices adapted from WDC-Kyoto on 4 February.

On 4 February, daytime enhancement in EEJ started from ~ 15:00 UT, there was a sharp decrease in EEJ between ~ 15:50 and 17:00 UT, which coincides with the decrease in dawn-to-dusk interplanetary electric field (IEFy). However, significant enhancement in EEJ afterward could be caused by another driver. To understand this, we studied the possibility of occurrence of substorm activity on 4 February. Figure 5a–c shows the variation of the EEJ and IEFy (top panel), GOES 17 Differential Proton Flux (middle panel), and auroral electrojet indices AU (amplitude upper) and AL (amplitude lower) (bottom panel) respectively on 4 February 2022. It should be noted out that AU/AL data are real-time preliminary data adapted from World Data Center for Geomagnetism in Kyoto (WDC‐Kyoto, https://wdc.kugi.kyoto-u.ac.jp/ae_realtime/) and thus, it may contain noise and baseline shift.

Initially, the variation of the eastward electric field closely followed the dawn-to-dusk electric field up to ~ 17:00 UT (indicated by the shaded region in Fig. 5a). The proton flux measured by the GOES-17 satellite showed sudden increases at ~ 15:00 UT, and ~ 17:40 UT (vertical dashed lines #1 and #2), indicating flux injections at substorm onsets (Fig. 5b). These well-defined structures with gradual decrease and sudden increase (sawtooth-like shape) represent the type of substorm termed as the sawtooth event. The presence of substorm events are also confirmed by strong disturbances in the AL indices. The AU and AL indices indicate the current intensity of the eastward and westward auroral electrojets. AL continued to grow and remain disturbed on 4 February, with maximum AL decreased at ~ 15:00 UT and ~ 18:14 UT (Fig. 5c). This might result in the enhanced eastward electric field at low-latitudes, due to the presence of sawtooth substorm events. Our results are consistent with the studies by Huang et al.^68,69^. They reported that under steady southward IMF Bz condition, the convection electric field and EEJ continue to increase at the equator during the expansion phase of magnetospheric substorms during the sawtooth event. Therefore, the strong enhancement in eastward current on 4 February with a maximum enhancement of ~ 158 nT at ~ 18:25 UT (vertical dashed line #3 in Fig. 5) could be attributed to enhanced magnetospheric convection due to the substorms onsets. This prevailing daytime eastward electric field explains the observed enhancement in TEC over the northern low-latitudes.

The MAGE simulated meridional wind direction reverses from equatorward to poleward at ~ 14:00 UT on 3 February and subsequently the equatorial electric field becomes eastward (Fig. 4). On 4 February, substorm onset at ~ 15:00 UT and 17:30 UT enhances the eastward field due to substorm convection. The substorms also induce an equatorward neutral wind at high-latitude that slowly propagates towards the low-latitudes in both hemispheres. However, MAGE model simulation shows that in low-mid latitudes, neutral wind direction is mostly nullified because of the presence of dominating daytime poleward wind from 15:00 to 20:00 UT. Therefore after 15:00 UT, the penetration electric field overrides the neutral wind effect. Thus, the significant enhancement in the daytime EEJ on 4 February is mainly attributed to the convection electric field during substorm.

Overall, the following features are noticed during the 3–4 February 2022 geomagnetic storm:

The storms caused strong morning counter electrojet (CEJ) (~ 9:00–14:30 UT) on 3 and 4 February. This could be caused by the DDE field generated by storm-induced equatorward thermospheric wind circulation (3 and 4 February) and overshielding conditions resulting from the abrupt northward turning of IMF after a prolonged southward IMF on 3 February. This was followed by a significant enhancement in the eastward electric field near the dip-equator (EEJ) on both days. This enhancement in eastward electric field could be related to the prompt penetration electric (PPE) field and magnetospheric convection related to substorms.

On 3 February, the storm caused enhancement in the daytime ionosphere near EIA crest latitudes and severe negative effect in high-latitude. Storm-time perturbation over low-latitudes were not so significant. Whilst, a minor enhancement was noticed during storm recovery period over SH near-crest and mid-high latitudes which is not a usual storm-time phenomena, which could be related to neutral density enhancement. Usually, the daytime enhancement over low-mid latitudes mainly caused by enhanced O/N_2_ and the presence of a strong afternoon EEJ which is a classic storm-time phenomena.

The GOLD-derived thermospheric column density ratio O/N_2_ (ΣO/N_2_) exhibits significant perturbation during this weak geomagnetic storm. A substantial enhancement in O/N_2_ was observed over NH (winter) low-mid latitudes and depletion in high-latitudes. Whereas, over the SH (summer), O/N_2_ enhancement was observed in the low-latitudes with an extended negative response (O/N_2_ depletion) over mid-high latitudes on 4 February. This hemispheric asymmetry observed in neutral composition can be explained in terms of combine effect of storm-time and seasonal changes/circulation that usually occurs during intense storm.

One of the novel storm-time characteristics observed during this minor storm is the unexpected/inconsistent variation between TEC (depleted) and O/N_2_ (enhanced) over a low-latitude stations. The strong CEJ on 4 February (before 16 UT) assists to explain it by suggesting that prenoon (before 16 UT) TEC variations were primarily controlled by the CEJ related electrodynamical changes driven by DDE field.

The generation of morning CEJ over the American sector due to disturbance dynamo electric field is explained by the MAGE model simulations. The model simulations demonstrate how storm-induced equatorward thermospheric wind circulation at high latitudes gradually generates a westward electric field at near-equatorial regions. Correspondingly, the GOLD-measured Tdisk temperature at high-mid latitudes also provide evidence of the generation of storm-induced equatorward thermospheric wind circulation at high latitudes.

The enhanced O/N_2_ and the presence of an intense afternoon EEJ contributed to a significant enhancement of ionospheric electron density over the NH low-latitude during daytime and afternoon enhancement over SH low-latitudes as observed from GPS-TEC measurements on 4 February. The severe negative ionospheric effect (TEC depletion) in NH high-latitude was mainly contributed by O/N_2_ depletion, which is well known. Thus, the afternoon ionospheric response over equatorial and low-latitude stations are contributed by both electrodynamical and compositional changes induced by geomagnetic storms. Whereas, over mid to high-latitudes, storm-time ionospheric changes are caused by neutral dynamical changes.

## Discussions

The high-to-low-latitude coupling during geomagnetic storms often modifies the ionospheric electron density distribution by producing positive ionospheric storm (enhanced electron density) and/or negative ionospheric storm (decreased electron density)^15,23,36,70–75^. These changes are mainly due to storm time modulations in ionospheric electric fields and currents (e.g., Nishida^76^; Sastri et al.^77^; Bagiya et al.^15^; Astafyeva et al.^36,37^; Singh et al.^26,36,37,76,77^) and/or thermospheric wind circulation and neutral compositional changes globally (e.g., Fuller-Rowell et al.^9^; Bagiya et al.^23^ and references therein). These effects can be severe depending on the intensity of the storm and the local time of the day^78,79^. For moderate to minor geomagnetic storms, it becomes rather challenging to delineate the I-T changes from the background variations. However, the study addressed in the present paper suggests that this is not the typical characteristic. Although the geomagnetic conditions of 3–4 February 2022 were identified as G1-class (minor) storms, it has caused significant changes in ionospheric electric fields and thermospheric neutral compositions in the dayside ionosphere.

The moderate to minor geomagnetic storms are frequent during declining and rising phases of the solar cycle, and their major driving forcing are high-speed solar wind streams (HSS), corotating interactive region (CIR), and slow-moving CMEs^80–83^. These reports showed that prompt penetration electric field (PPEF) variations during CIR-driven weak and moderate geomagnetic storms could also induce substantial modifications in EEJ with response to the changes in IEFy. It is well understood that PPEF at the equatorial ionosphere is proportional to IEFy^22,84,85^*.* During the southward turning of IMF Bz, the penetration of convection field from high to low-latitudes manifests as PPEF. The PPEF effect is eastward in the dayside ionosphere, which enhances the ionospheric electric fields and, thus upward drift of plasma. The increase in upward drift raises the plasma to higher altitudes in a region of lesser recombination rate, which ultimately increases electron density. A sudden northward turning of IMF Bz gives rise to a strong westward electric field at low latitudes resulting in CEJ. The overshielding condition during the northward turning of IMF Bz could result in the westward electric field^14,84,86–89^. This overshielding condition develops rapidly, responding to solar wind conditions. The appearance of CEJ over the American low latitudes on 3 February coincides with the northward turning of IMF Bz. Another cause of CEJ is the delayed effect of disturbance dynamo^8^, which is activated in the mid-latitude thermosphere/ionosphere by the disturbed equatorward wind from the polar thermosphere. In the present study, we use the MAGE simulations to elucidate the development of DDE field. Recently, Laskar et al.^90^ and Lin et al.^65^ compared and validated the MAGE simulations with other models like TIE-GCM and empirical NRLMSIS model outputs and found that MAGE performs better in predicting the neutral density variation during this minor storm. The MAGE simulations showed that the generation of strong equatorward meridional wind from high-latitudes and downward vertical drift generates a strong westward field near the equator. The disturbance dynamo initiates with a time lag of several hours from the commencement of the storm and continues for another several hours^71^. However, it should be noted that the CEJ that occurs during the storm main-phase could also be possibly caused by the DDE field activated by the preceding storm activities. The electrodynamic conditions on 3 February could not reflect in the TEC variations over SH. The thermospheric compositional changes probably masked the same. Nevertheless, morning-to-noon depletion in TEC on 4 February over low-latitude stations was attributed to CEJ, although there was an enhancement in the O/N_2_ density. The significant daytime TEC enhancement on 3 and 4 February over NH low-latitude station BOGT coincides with the EEJ enhancement. Therefore, the low-latitude electric field variations due to the PPEF and magnetospheric convection during substorm onset contributed significantly towards generating positive storm over the dayside low-latitude ionosphere.

The ionospheric response during the storm recovery phase is rather a complex process characterized by long-duration negative^23,39,91^ or positive^74,92^ effect depending on the latitude, longitude, and intensity of the storm. The I-T response to the minor geomagnetic storm of 3–4 February shows distinct interhemispheric asymmetry, which is attributed to the combined effects of storm-induced and seasonal wind circulation patterns. The occurrence of positive and negative storms also shows seasonal dependency mainly caused by the storm-time compositional changes in the neutral atmosphere^9,93^*.* It has been well reported that positive storms are most common in the winter hemisphere, and negative storms are most common in the summer hemisphere. The present study showed that weak geomagnetic storms could also cause strong seasonal hemispheric asymmetry characterized by a strong negative impact in summer (SH) and a prevalent positive impact in winter (NH). Recent studies by Cai et al.^61,94^ reported significant perturbations in the thermospheric O/N_2_ during minor geomagnetic storms. Cai et al.^61^ using GOLD measurements, showed that during minor storms that occurred in May and June 2019 (northern summer), the upward vertical winds amplified after the onset of the storm led to a reduction in ΣO/N_2_ at the high-latitudes. This depleted O/N_2_ region (compositional bulge) is initially formed by the heating and upwelling of air in the auroral region, then carried equatorward by strong meridional winds during summer^95^*.* The observed extended depletion in ΣO/N_2_ over the SH mid-high latitudes on 4 February 2022 can be explained by this mechanism. MAGE simulations showed that the equatorward wind still persisted throughout the day on 4 February over mid-latitudes which was not the case on 3 February (Fig. 4). Furthermore, Cai et al.^62^ found a comparatively enhanced O/N_2_ region (the neutral tongue) sandwiched by two depleted regions over North America and the Atlantic Ocean during a minor geomagnetic storm that occurred in May 2019. By using TIE-GCM simulations, they showed that the formation of this structure was attributed to a change in neutral wind direction that transported the O/N_2_ to that longitude sector. Additionally, our study shows strong morning-afternoon asymmetry in ionospheric and thermospheric perturbations during storm. Recently, Laskar et al.^64^ studied the local time variation of thermospheric temperature during the geomagnetically disturbed period using GOLD neutral disk temperature (Tdisk) data. They reported morning-afternoon differences in Tdisk data during geomagnetic storm, which is the first experimental evidence of numerical model simulation by Burns et al.^96^. They found morning enhancement in temperature is more than afternoon. This is consistent with our results that shows strong ΣO/N_2_ and Tdisk enhancement during the morning over SH low-latitudes.

Furthermore, on 4 February, the SH low-latitude enhancement in O/N_2_ is not reflected in the TEC (morning to noon hours), which is an unexpected behavior. The observed morning depletion over the low-latitude regions (particularly over IQQE) can be attributed to the presence of the CEJ event as discussed previously (Fig. 3 and 4). Furthermore, over the winter hemisphere, the compositional bulge is confined at the high-latitudes as it is restricted by the daytime poleward wind. MAGE simulation on 3 February shows strong poleward wind after 14 UT. Thus there is a decrease in the molecular species (N_2_) resulting in the observed enhanced ΣO/N_2_ and thus the enhanced electron density at NH low-mid latitudes (winter hemisphere). Therefore, the changes in the thermospheric neutral composition at the low-latitudes during the storm are attributed to the storm time circulation changes and along with the modification in seasonal wind patterns.

## Conclusions

The present investigation is first of its kind in exploring the dayside I-T response to G1- class minor storm activity that occurred during 3–4 February 2022 using a suite of ground and space based ionospheric, thermospheric observations, and model simulation. The overall conclusion is minor storms can also produce multifaceted effects in the ionosphere-thermosphere system. Therefore, it is suggested that studies related to minor storm effects on the terrestrial atmosphere requires more attention. The major conclusions of these studies are summarized as follows:

- Results revealed that G1-class geomagnetic storm could also cause substantial enhancement (> ~ 100%) in TEC at the low-mid latitudes, which is attributed to enhanced O/N_2_ and intense EEJ variation at the low latitudes and depletion over the mid-high latitudes (~ − 80%) caused by O/N_2_ depletion.
- An unexpected feature is revealed from the study, i.e. morning to noon increase in O/N_2_ and depletion in TEC over the American low-latitudes. This can be explained by the presence of a morning CEJ event caused by the disturbance dynamo effect. Therefore, the storm time electrodynamical modifications played a major role in inducing ionospheric variation over the American low-latitude stations.
- A strong morning to noon negative ionospheric response is observed over the SH mid-high latitudes, mainly attributed to the O/N_2_ depletion. Thus, over mid to high-latitudes, the storm time electrodynamical effects were overridden by neutral dynamical changes driven by storm induced circulation.
- Substantial enhancement in daytime EEJ strength in the afternoon induces positive effect in TEC over low-latitudes. Low-latitude ionospheric electric field/EEJ variation on 4 February could be related to the DDE, PPE field, and magnetospheric convection related to the substorm.

Thus, the combined effect of the storm-induced neutral dynamic and electrodynamic forcing along with the effect of seasonal circulation controlled the dayside I-T system during these weak geomagnetic storms.

## Methodology

### Solar and interplanetary parameters, geomagnetic index, and proton flux

For the present work, the 1-min high-resolution plasma and magnetic field parameters measured by ACE spacecraft at the L1 point are used to describe the interplanetary conditions. The solar wind velocity (SW), northward/southward component of Interplanetary magnetic field (IMF Bz), Flow pressure, Electric field (IEFy), Symmetric H-component (SYM-H) index data are obtained from http://cdaweb.gsfc.nasa.gov. IEFy indicates the dawn-to-dusk (y-component**)** interplanetary electric field calculated using IMF Bz and velocity Vx*.*

The proton flux data measured by the Space Environment In Situ Suite (SEISS) onboard GOES-17 are used to study the energetic plasma flux injections at substorm onsets. This data is available at https://www.ngdc.noaa.gov. SEISS is a suite of particle detectors flown on the GOES-R Series (GOES 16/17) that measures the plasma properties and energetic particle populations in geosynchronous orbit^97^.

### GPS-TEC data

The GPS measured TEC served as main database to study the ionospheric variations addressed in this study. TEC are calculated from GPS measurements over the American longitudes. The locations of these stations are shown in Supplementary Fig. S1 and the geographic and magnetic coordinates of these stations are presented in Supplementary Table S1.

These GPS stations are part of the International GNSS Service (IGS) network, and related data at 30-s resolution are obtained from Crustal Dynamics Data Information System (CDDIS DAAC). The GPS raw measurements of differential code (pseudo range) and carrier phase from Receiver INdependent EXchange **(**RINEX) files were used to derive slant total electron content (sTEC). The detailed methodology for deriving sTEC can be found in Calais & Minster^98^. Elevation mask of 30° is applied while deriving sTEC. The sTEC is converted to vertical TEC (vTEC) by taking the vertical projection following the method suggested by Klobuchar^99^. The vTEC was further subjected to the 1-min moving average.

The vTEC map shown in Supplementary Fig. S2 were obtained from Massachusetts Institute of Technology (MIT)’s Haystack Observatory Madrigal database (http://cedar.openmadrigal.org/), which provides global VTEC maps with 5-min temporal resolution and 1 × 1° spatial resolution in latitude and longitude.

### Magnetometer data

EEJ is used as a proxy to learn the low-latitude ionospheric electric field variations during geomagnetic storms^100,101^. To understand the low-latitude electric field variations over the American sector, magnetometer data from the Geophysical Institute of Perú (IGP) network are obtained from the Low Latitude Ionospheric Sensor Network (LISN) (http://lisn.igp.gob.pe). EEJ strength was derived by using Δ*H*(*Jicamarca*, *equator*) − Δ*H*(*Tarapoto*, *off-equator*). Respective ΔH is derived from the perturbations in horizontal component of the geomagnetic field (H) at Jicamarca (11.95° S, 76.87° W) and Tarapoto (6.49° S, 76.35° W). The nighttime baseline values are removed from H measurements for both stations in order to calculate ΔH.

### GOLD data

Global-scale Observations of the Limb and Disk (GOLD) is a NASA mission designed to measure the densities and temperatures in Earth's thermosphere and ionosphere. A Far-ultraviolet (FUV) imaging spectrograph on a geostationary satellite located in a geostationary orbit over 47.5°W is used for imaging the Earth's airglow from ∼ 134 to 162 nm. In daytime mode, the GOLD imager scans a maximum longitude range from 120°W to 20°E and a latitude range of 70°S–70°N. More details on GOLD payloads can be found elsewhere^102^. The GOLD mission data products include daytime brightness of the FUV emissions, neutral temperatures (Tdisk), atomic oxygen to molecular nitrogen column density ratio (O/N_2_), and the nighttime oxygen emission intensities on the disk^103^.

The O/N_2,_ also referred to as ΣO/N_2_, indicates the abundance of thermospheric compositions O and N_2_. It is derived from GOLD daytime disk scan (DAY) measurement. The ratio of the OI 135.6 nm and N_2_ Lyman-Birge-Hopfield (LBH) band intensities measured by GOLD on the dayside disk are used to retrieve the O/N_2_ ratio. The ratio of the vertical column density of O and N_2_ (ΣO/N_2_) calculated at a standard reference N_2_ depth of 10^17^ cm^−2^ as a function of the solar zenith angle (details in Correira et al.^63^)*.* Similarly GOLD daytime disk measurements are used to derive the disk temperature (Tdisk) which defines the effective disk neutral temperature at a height of approximately 150 km. The retrieval algorithm of Tdisk is an extension of the previously used method for the derivation of the temperature from limb measurements of LBH intensity from the High-resolution Ionospheric and Thermospheric Spectrograph (HITS) instrument^104^. The GOLD observations have a higher signal-to-noise ratio than HITS. The effective neutral temperatures are processed by fitting the observed rotational structure of the N_2_ LBH bands using an optimal estimation routine (details in Evans et al.^105^). O/N_2_ and Tdisk files were created by binning 2 × 2 Level 1C pixel and a single file is created at the end of the day, combining all individual files of a given day. These daily summary files are publicly released as L2 data products. The resulting O/N_2_ and Tdisk data product has a spatial (horizontal) resolution of 250 km × 250 km at the spacecraft nadir. Full-disk DAY scans begin at 03:00 satellite local time (06:10 UT), and it takes about 30 min to scan the whole disk. During the current study period, the scan times are 08:10 UT–18:22 UT at 2 h cadence. For the current study, we are using GOLD scan from 12:10 UT to 18:10 UT for NH (12:22–18:22 UT for SH) at 2 h intervals for maximum coverage. The northern and southern hemispheres are scanned separately, with each hemisphere scanning last about 12 min. The random and systematic uncertainties of GOLD ΣO/N_2_ data are ~ 5 and ~ 5%, respectively^63^. Typical random errors in the version 4 Tdisk data varies with signal to noise ratio of the N_2_ LBH emission and it ranges from 20 K (for high SNR) to tens of K (for low SNR)^5,64^.

### MAGE model data

MAGE model is a newly developed fully coupled whole geospace model. The MAGE consists of the Grid Agnostic MHD with Extended Research Applications (GAMERA) global MHD model of the magnetosphere^106,107^, the Rice Convection Model (RCM) of the ring current^108^, Thermosphere-Ionosphere Electrodynamics General Circulation Model (TIE-GCM) of the upper atmosphere^109^, and Redeveloped Magnetosphere-Ionosphere Coupler/Solver (REMIX)^110^. The MAGE model has been used to study atmospheric and ionospheric disturbances during storms^5,111–113^. The thermosphere-ionosphere part of the MAGE comes from TIEGCM. The TIEGCM is a time‐dependent, three‐dimensional model that self-consistently solves the fully coupled, nonlinear, thermodynamic, hydrodynamic, and continuity equations of the neutral gas, the ion and electron energy and momentum, and ion continuity equations and neutral wind dynamo from ~ 97 to 600 km^109^. The input parameters for the TIEGCM model are solar EUV and UV fluxes, auroral particle precipitation, an imposed magnetospheric electric field, and the amplitudes and phases of tides from the lower atmosphere. The ionospheric convection and auroral precipitation are specified from the MAGE suite. The TIE-GCM horizontal resolution in this case is 1.25° × 1.25° and vertical resolution is 0.25 scale height. For the present investigation only the meridional winds from MAGE are used.

### Supplementary Information

Supplementary Information.

## Data Availability

GPS observations measured at every 30-s GPS observations are obtain from https://cddis.nasa.gov. GOLD ΣO/N_2_ data are obtained from https://gold.cs.ucf.edu/. The magnetometer data at Jicamarca and Tarapoto under IGP network are collected from the website http://lisn.igp.gob.pe. The proton flux data are obtained from level 2 GOES 17 satellite’s Space Environment In Situ Suite (SEISS) data and are publicly available at https://www.ngdc.noaa.gov/stp/satellite/goes-r.html. Solar wind and interplanetary parameters as observed by ACE spacecraft are available at http://cdaweb.gsfc.nasa.gov/istp_public/. The TEC maps were obtained from MIT’s Madrigal database available at http://cedar.openmadrigal.org/.
