# Supplementary material for: Unveiling the combined effects of neutral dynamics and electrodynamic forcing on dayside ionosphere during the 3–4 February 2022 “SpaceX” geomagnetic storms
Source: Sci Rep. 2023 Nov 2;13:18932. doi: 10.1038/s41598-023-45900-y (PMC10622547; doi:10.1038/s41598-023-45900-y)
Supplement: Supplementary file 1 — Supplementary Information. [file 41598_2023_45900_MOESM1_ESM.docx]

Supporting Information for

**Unveiling the Combined Effects of Neutral Dynamics and Electrodynamic Forcing on Dayside Ionosphere during the 3 - 4 February 2022 “SpaceX” Geomagnetic Storms**

**Geetashree Kakoti^1*^, Mala S. Bagiya^1^, Fazlul I. Laskar^2^, and Dong Lin^3^**

^1^Indian Institute of Geomagnetism, Navi Mumbai, India

^2^Laboratory for Atmospheric and Space Physics, University of Colorado, Boulder, CO, USA

^3^High Altitude Observatory, National Center for Atmospheric Research, Boulder, CO, USA

**Contents of this file**

Supplementary Table S1

Supplementary Figures S1, S2, S3

**Introduction**

[The supplementary material consists of Table S1 showing coordinates of GPS stations over American sector used for the current study, Supplementary Figure S1 showing GPS-TEC stations, Supplementary Figure S2 depicting the % deviation of TEC from quiet day average TEC, Supplementary Figure S3 presents local time variation of GOLD %diff Tdisk (% deviation of Tdisk temperature on storm days from quiet day) for different latitudinal sectors]

**Text S1**.

The geographic and magnetic coordinates of the GPS station locations over American longitude are presented in Table S1. The GPS stations of KUJ2, GODZ, BOGT, and RIOP are in the northern hemisphere (NH), and IQQE, CHPI, ANTC, and FALK are in the southern hemisphere (SH). The RIOP, IQQE, BOGT, CHPI, and are near-equatorial to low-latitude stations; ANTC, GODZ, FALK, and KUJ2 are mid to high latitude stations.

**Supplementary Table S1. GPS stations with their geographic coordinates, magnetic dip, and geomagnetic latitude.**

| **Stations** | **Geographic Latitude and Longitude** | **Magnetic Dip** | **Geomagnetic Latitude** |
| --- | --- | --- | --- |
| **KUJ2** | 55.29°N,77.73°W | 76.87° dip | 64.52°N |
| **GODZ** | 39.02°N, 76.83°W | 65.19° dip | 48.2°N |
| **BOGT** | 4.64°N, 74.08°W | 26.98° dip | 14°N |
| **RIOP** | 1.65°S, 78.65°W | 17.7° dip | 7.67°N |
| **IQQE** | 20.27°S, 70.13°W | -17.94° dip | 10.84°S |
| **CHPI** | 22.6°S, 44.9°W | -39.57° dip | 14.26°S |
| **ANTC** | 37.33°S, 72°W | -38.8° dip | 28°S |
| **FALK** | 51.69°S, 57.87°W | -50.88° dip | 42.65°S |


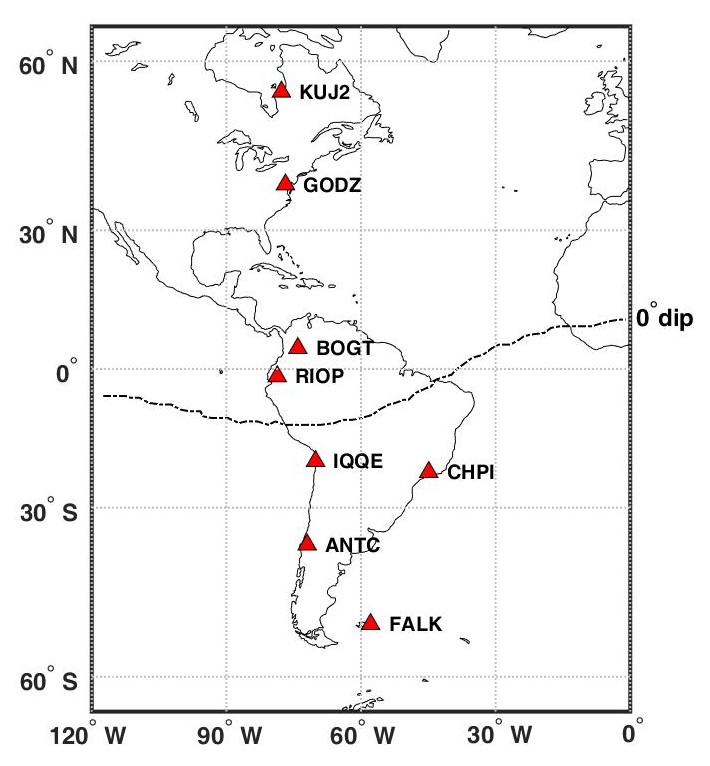


**Supplementary Figure S1. Map showing locations (red triangles) of the GPS stations used in this study.** **The map is generated by using MATLAB® (Release 2023a)** (URL: https://in.mathworks.com/products/new_products/release2023a.html).


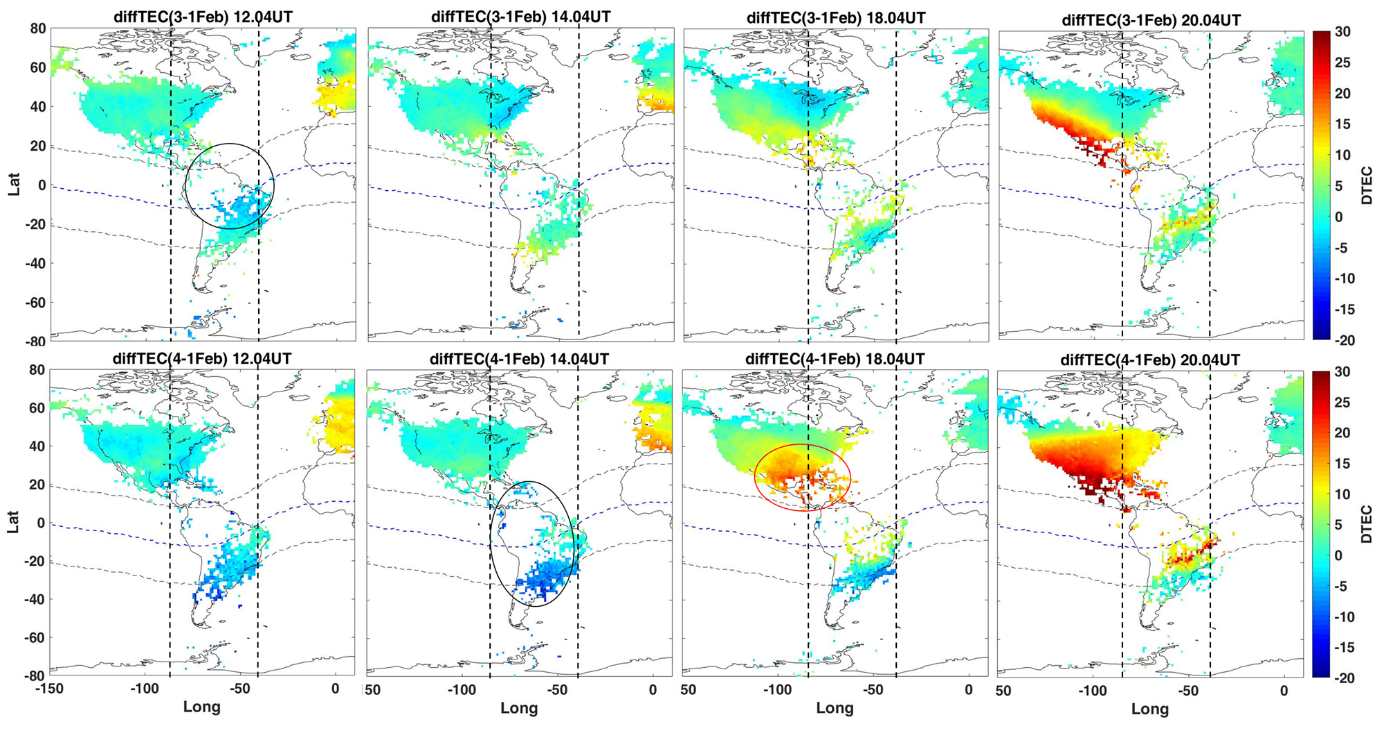


**Supplementary Figure S2. Map of dTEC variation over American longitude for ~12 and 14 UT (morning) and 18 and 20 UT (afternoon) on 3 (top panel) and 4 (bottom panel) February 2022. TEC enhancement/depletion are marked by red/black circles.** **The map is generated by using MATLAB® (Release 2023a)** (URL: https://in.mathworks.com/products/new_products/release2023a.html).


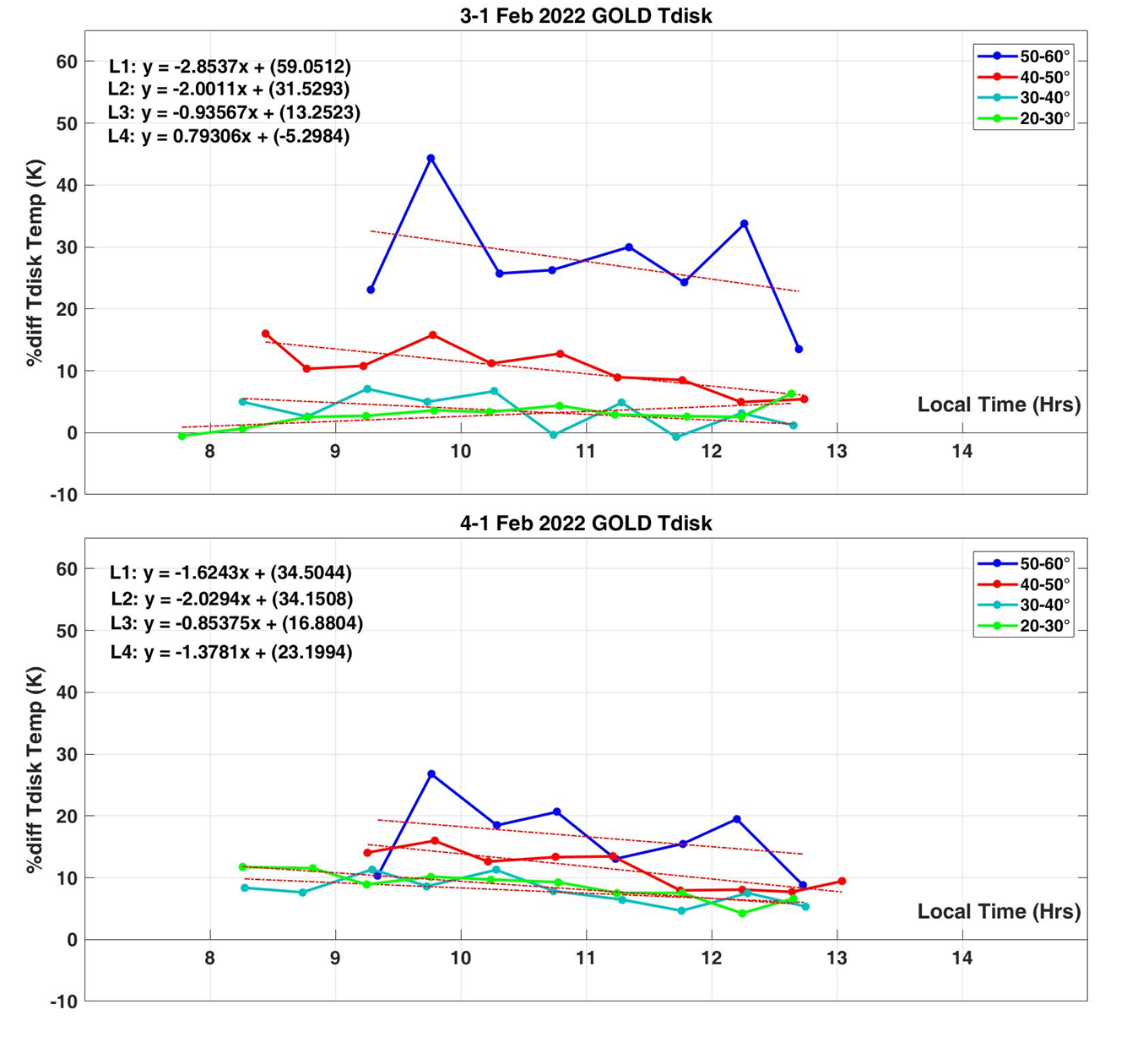


**Supplementary Figure S3. Local time variation of percentage change in GOLD Tdisk temperature (%Diff Tdisk) over American longitude on 3 (top panel) and 4 (bottom panel) February 2022 from quiet day (1 February 2022) for different latitudinal sectors.**
